# Retraction Note: Diacerhein attenuates the inflammatory response and improves survival in a model of severe sepsis

**DOI:** 10.1186/s13054-016-1453-8

**Published:** 2016-09-01

**Authors:** Kelly L Calisto, Angélica C Camacho, Francine C Mittestainer, Bruno M Carvalho, Dioze Guadagnini, José B Carvalheira, Mario J Saad

**Affiliations:** Department of Internal Medicine, FCM, State University of Campinas (UNICAMP), Cidade Universitária Zeferino Vaz, Campinas, SP Brazil

The authors would like to retract this article [1] because it was brought to the Editors’ attention that some figures appear to be similar to those within the article and in previous publications [2–11]. The similarity between the figures within this article and previous publications, are specified below. An investigation by The University of Campinas (UNICAMP) in São Paulo, Brazil concluded that there was no evidence of research misconduct. The authors maintain that the similarities do not affect the interpretation or conclusions of their study. The authors accept that the preparation of the figures fell below the standard of publication. They will seek to publish the results in a new manuscript version, with new experiments and figures, corroborating the findings of this work. Author, Jose Carvalheira agreed to the retraction and did not disagree with the wording of the retraction.

Figure 4B (IRS-1) from [1] and Fig. 4C (b-actin) from the same article [1].

Figure 4B (IB:IR) from [1]:

And Fig. 1D (IB:IKKb) from Flores et al. 2012 [2]

And Fig. 2D (IB:IRb) from Flores et al. 2012 [2]

And Fig. 4I (IB: GLUT4) from Prada et al. 2006 [3]

And Fig. 6F (IP:IR/IB:pY) from Flores et al. 2006 [4]

And Fig. 2B (Total AMPK) from Ropelle et al. 2007 [5]

And Fig. 3B (Total p70S6K) from Ropelle et al. 2008 [6]

And Fig. 2C (IB: a-Tubulin) from Ropelle et al. 2009 [7]

And Fig. 2G (IB: JAK2) from Ropelle et al. 2008 [8]

And Fig. 2H (IB: STAT3) from Ropelle et al. 2008 [8]

Figure 4B (IB: AKT) from [1]:

And Fig. 1C (IB: IRS2) from De Souza et al. 2010 [9]

And Fig. 1G (IB: Foxo1) from De Souza et al. 2010 [9]

And Fig. 4C (IB: IRS1) from Prada et al. 2006 [3]

And Fig. 2D (Total AMPK) from Ropelle et al. 2007 [5]

And Fig. 2F (a-Tubulin) from Ropelle et al. 2007 [5]

And Fig. 3A (IB: IKKb) from Oliveira et al. 2011 [10]

And Fig. 6L (Total eIF4E) from Ropelle et al. 2008 [6]

Figure 4C (IB: IRS-1) from [1], lanes 3 – 4 and 7-8

Figure 4C (IB: IRS-1) from [1] lane 5 and lane 6

Figure 5A (IB: p-KKb) from [1], and Fig. 5E (IB: INOS) from Calisto et al. 2010 [11]

Figure 5A (IB: b-Actin) from [1],

And Fig. 6A (IB: b-Actin) from the same article [1],

And Fig. 7B (IB: b-Actin) from the same article [1]

And Fig. 4I (IB: IRS1) from Calisto et al. 2010 [11]

Figure 5B (IB: b-Actin) from [1]:

And Fig. 6C (IB: b-Actin) from the same article [1]

And Fig 4G (IB: IRS1) from Calisto et al. 2010 [11]

And Fig. 6D (IB: b-Actin) from Calisto et al. 2010 [11]

Figure 5B (IB: NF-kB) from [1] and Fig. 4I (IB: p-IRS1Ser307) from Calisto et al. 2010 [11]

Figure 5C (IB: NF-kB) from [1] and Fig. 3E (IB: pIKK) from Calisto et al. 2010 [11]

Figure 7B (IB: p-PERK) from [1] and Fig. 5F (IB: INOS) from Calisto et al. 2010 [11]

Figure 7C (IB: b-Actin) from [1] and Fig. 4H (IB: IRS1) from Calisto et al. 2010 [11]
